# Dual EGFR and ABL Tyrosine Kinase Inhibitor Treatment in a Patient with Concomitant EGFR-Mutated Lung Adenocarcinoma and BCR-ABL1-Positive CML

**DOI:** 10.1155/2020/4201727

**Published:** 2020-03-19

**Authors:** Kousuke Watanabe, Hidenori Kage, Saki Nagoshi, Kazuhiro Toyama, Yoshiyuki Ohno, Aya Shinozaki-Ushiku, Kumi Nakazaki, Hiroshi Suzuki, Mineo Kurokawa, Takahide Nagase

**Affiliations:** ^1^Department of Respiratory Medicine, The University of Tokyo, Tokyo, Japan; ^2^Department of Hematology and Oncology, The University of Tokyo, Tokyo, Japan; ^3^Department of Pharmacy, The University of Tokyo Hospital, Faculty of Medicine, The University of Tokyo, Japan; ^4^Department of Pathology, The University of Tokyo, Tokyo, Japan

## Abstract

Tyrosine kinase inhibitor (TKI) combination is expected to increase in the era of precision medicine. TKI combination may be required to treat double primary cancers, each having a targetable gene, or to treat a single malignancy with multiple targetable genes. Here, we demonstrate the first report of dual EGFR and ABL TKI treatment in a patient with concomitant EGFR-mutated lung adenocarcinoma and BCR-ABL1-positive chronic myeloid leukemia (CML). A 60-year-old man with an 8-year history of CML was diagnosed as advanced EGFR-mutated lung adenocarcinoma. Complete molecular response of CML had been achieved by imatinib, and ABL-TKI had been switched to nilotinib four years previously due to muscle cramps. We discontinued nilotinib and started afatinib. Although partial response of lung adenocarcinoma was achieved, cytogenetic relapse of CML was observed following nilotinib discontinuation. We applied the previously described framework of cytochrome P450 3A4-mediated oral drug-drug interactions and selected gefitinib and nilotinib to treat both malignancies. We effectively and safely administered this combination for seven months. The present report is the first to demonstrate the safety and efficacy of dual EGFR and ABL TKI treatment in a patient with concomitant EGFR-mutated lung adenocarcinoma and CML.

## 1. Introduction

The prognosis of chronic myeloid leukemia (CML) has been dramatically improved by ABL tyrosine kinase inhibitor (TKI), with 5- and 8-year overall survival rate of 90% and 88%, respectively [1]. The prognosis of advanced EGFR-mutated lung adenocarcinoma has also been improved by EGFR-TKI, and the median progression free survival in the first-line setting is 10-12 months for the first and the second generation EGFR-TKIs and 18.9 months for the third generation EGFR-TKI osimertinib [2, 3].

TKI combination therapy is expected to increase in the era of precision medicine for two reasons. First, the cumulative incidence of a new primary malignancy is expected to increase with improved prognosis of the first malignancy, and TKI combination may be required to treat both malignancies. Second, clinical sequencing will uncover multiple targetable genes in a single tumor, and TKI combination is currently being investigated as a rational treatment strategy to overcome TKI resistance [4].

Here, we report dual EGFR and ABL tyrosine kinase inhibitor treatment in a patient with advanced EGFR-mutated lung adenocarcinoma with an 8-year history of BCR-ABL1-positive CML. Limited information is available on the combination of EGFR-TKI and ABL-TKI, and the drug-drug interaction between EGFR-TKI and ABL-TKI is of considerable importance for the concomitant use of both TKIs.

We have previously established a quantitative prediction framework of cytochrome P450 (CYP) 3A4-mediated oral drug-drug interactions [5, 6], and this method was applied to predict the drug-drug interaction between EGFR-TKI and ABL-TKI. Using this method, the increase of an area under the concentration-time curve (AUC) of CYP3A4 substrate by a CYP3A4 inhibitor can be calculated using the equation 1/(1 − CR_CYP3A4_ × IR_CYP3A4_), where CR_CYP3A4_ is the ratio of contribution of CYP3A4 to clearance of a substrate drug after oral absorption and IR_CYP3A4_ is the time-averaged apparent inhibition ratio of the inhibitor. The CR_CYP3A4_ of a substrate is calculated based on the AUC increase observed in interaction studies with typical CYP3A4 inhibitors, such as ketoconazole and itraconazole. The IR_CYP3A4_ of an inhibitor is calculated based on the AUC increase of standard CYP3A4 substrate, such as midazolam. This framework can be applied to predict the magnitude of unknown CYP3A4-mediated drug-drug interactions.

The combination of gefitinib and nilotinib was selected based on the prediction of drug-drug interaction, and the patient was safely treated by the combination for seven months. As far as we know, this is the first report of dual EGFR and ABL tyrosine kinase inhibitor treatment for concomitant EGFR-mutated lung adenocarcinoma and BCR-ABL1-positive CML. The present case also demonstrates the usefulness of our prediction framework for CYP3A4-mediated drug-drug interactions in cancer therapeutics in general.

## 2. Case Presentation

A 60-year-old man with a 21-pack-year history of tobacco use presented with hoarseness due to left recurrent laryngeal nerve palsy. He had been diagnosed as BCR-ABL1-positive CML eight years previously and had achieved a complete molecular response using imatinib. The ABL-TKI had been switched to nilotinib four years previously due to imatinib-induced muscle cramps without relapse.

**Figure 1:**
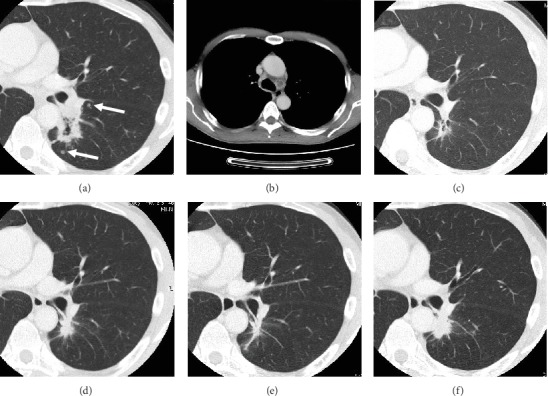
Chest CT scan. Chest CT scan performed on admission (a, b) four months after afatinib treatment (c) before the initiation of gefitinib (d) two months after gefitinib treatment (e) and six months after gefitinib treatment (f). Arrows indicate separate tumor nodules in the same lobe as the primary tumor.

A computed tomography (CT) was performed to investigate the cause of the recurrent laryngeal nerve palsy and revealed a 4 cm cavitary pulmonary mass in the left lower lobe with mediastinal lymph node swelling (Figures 1(a) and 1(b)). Physical examination, complete blood cell count, and blood chemistry studies revealed no remarkable findings, while serum CEA level was elevated to 19.1 ng/ml.

**Figure 2:**
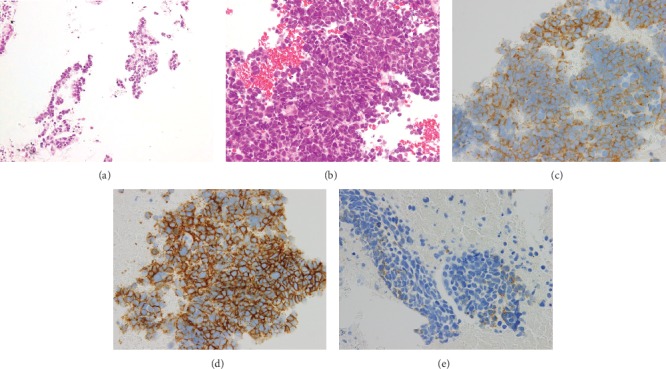
Microscopic features of lung cancer. (a) Adenocarcinoma cells from the first biopsy of a mediastinal lymph node. (b) Small cell transformation from the second biopsy of the hilar lymph node (hematoxylin and eosin stain; original magnifications: (a) ×200; (b) ×400). (c–e) Immunohistochemistry of small cell carcinoma cells with neuroendocrine markers. Synaptophysin (c), CD56 (d), and chromogranin A (e) (c–e, ×400).

Endobronchial ultrasonography-guided transbronchial needle aspiration of the mediastinal lymph node revealed adenocarcinoma cells (Figure 2(a)). The tumor was positive for thyroid transcription factor 1 (TTF-1) and EGFR mutation (exon 19 deletion), and a magnetic resonance imaging (MRI) revealed a single bone metastasis in the second lumbar vertebra. Separate tumor nodules in the left lower lobe were also noted by a chest CT scan (Figure 1(a)), and the patient was diagnosed as stage IVA (cT3N3M1b) lung adenocarcinoma.

**Figure 3:**
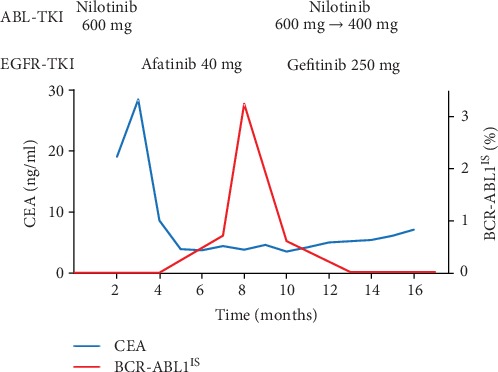
Serum CEA levels and BCR-ABL1 transcript levels on the international scale (BCR-ABL1^IS^) during the treatment.

Because the patient had maintained major molecular response of CML and no case report had ever described the combination of EGFR-TKI and nilotinib, we discontinued nilotinib and started treatment with afatinib (Figure 3). After four months of afatinib treatment, partial response of the lung adenocarcinoma was confirmed by a CT scan (Figure 1(c)) with decreased CEA level (Figure 3). However, blood BCR-ABL1 level on the international scale (BCR-ABL1^IS^) measured by the real-time quantitative reverse transcriptase polymerase chain reaction increased four months after the discontinuation of nilotinib (Figure 3). As the patient lost complete cytogenetic response (BCR-ABL1^IS^ increased to 3.2426%, and the percentage of BCR-ABL1-positive cells by fluorescence in situ hybridization of peripheral neutrophils was 13%), we discontinued afatinib and restarted nilotinib five months after its discontinuation.

At the time, three EGFR-TKIs (gefitinib, erlotinib, and afatinib) were approved in Japan as the first-line treatment of EGFR-mutant lung cancer; thus, pharmacokinetic drug-drug interaction between each EGFR-TKI and nilotinib was considered. Estimating the change in plasma concentrations of nilotinib and afatinib when given together was difficult, as both are substrates and inhibitors of P-glycoprotein (P-gp) [7–10]. Gefitinib and erlotinib are substrates for CYP3A4 [11, 12], and we predicted the increase of their plasma concentrations when coadministered with nilotinib, an inhibitor for CYP3A4, using the previously described quantitative prediction framework [5, 6]. The CR_CYP3A4_ of EGFR-TKIs were calculated based on the AUC increases reported in interaction studies with itraconazole or ketoconazole [13–16] (Table 1). The IR_CYP3A4_ of nilotinib was calculated to be 0.67 based on the 2.6-fold AUC increase of CYP3A4 substrate midazolam [17]. Using the equation 1/(1 − CR_CYP3A4_ × IR_CYP3A4_), the AUCs of gefitinib and erlotinib were predicted to increase by 1.44- and 1.36-fold, respectively.

After the approval by the institutional review board, the combination therapy by gefitinib and nilotinib was initiated (Figure 3). Gefitinib was selected for the combination with nilotinib, because the recommended dose of gefitinib (250 mg per day) is one-third of the maximum tolerated dose [18], whereas the recommended dose for erlotinib (150 mg per day) is the maximum tolerated dose [19]. Nilotinib is also a substrate of CYP3A4, and its CR_CYP3A4_ was calculated to be 0.67 based on its 3-fold AUC increase by CYP3A4 inhibitor ketoconazole [20]. Although gefitinib has not been shown to inhibit CYP3A4, the dose of nilotinib was decreased from 600 mg to 400 mg per day to avoid unexpected side effects of the TKI combination.

The primary lung tumor increased in size after discontinuation of afatinib (Figure 1(d)) but decreased again two months after gefitinib (Figure 1(e)). Major molecular response of CML was achieved five months after the readministration of nilotinib. The combination of gefitinib and nilotinib was safely administered for seven months. Only grade 1 skin rash and diarrhea were observed during the TKI combination according to the Common Terminology Criteria for Adverse Events (CTCAE) version 5 [21]. Although major molecular response of CML was sustained, the lung adenocarcinoma became resistant to gefitinib six months after the combination therapy (Figure 1(f)). The rebiopsy of the left hilar lymph node revealed small cell transformation without secondary EGFR T790M mutation (Figure 2(b)). The small cell carcinoma cells were diffusely positive for synaptophysin and CD56 and focally positive for chromogranin A (Figures 2(c)–2(e)).

## 3. Discussion

CML is a myeloproliferative neoplasm characterized by BCR-ABL1 fusion gene. The prognosis of CML has been dramatically improved by ABL-TKI, and the occurrence of a new primary malignancy can be a serious problem during the clinical course. There are limited case reports of concomitant EGFR-mutated lung adenocarcinoma and CML. Kaneshiro et al. report a case of recurrent EGFR-mutated lung adenocarcinoma in a patient receiving nilotinib for CML. In that report, nilotinib was discontinued, and the patient was successfully treated by gefitinib monotherapy without CML relapse [22]. To our knowledge, the present case is the first report of the dual EGFR and ABL TKI treatment in a patient with concomitant EGFR-mutated lung adenocarcinoma and BCR-ABL1-positive CML.

Data are conflicting whether the incidence of second malignancy is increased or decreased in CML patients receiving ABL-TKI. Verma et al. report that the risk of secondary malignancy was lower than expected in 1445 patients treated with ABL-TKI [23]. A study based on the Swedish CML registry reports increased incidence of a second malignancy with a standardized incidence ratio of 1.52 [24]. Data from cancer registries in Japan show that the incidence of a second malignancy is the same as that in the general population [25].

No standard therapy exists in advanced lung cancer patients with myeloid malignancy. The combination of dasatinib with EGFR-TKIs erlotinib or gefitinib has been reported as phase I/II clinical trials for advanced non-small-cell lung cancer [26, 27]. However, there has been no report on the concomitant use of EGFR-TKI and ABL-TKI in a patient with concomitant lung adenocarcinoma and CML, and only the alternating erlotinib and imatinib therapy has been reported in a case of concomitant EGFR-mutated lung adenocarcinoma and c-kit-mutated gastrointestinal stromal tumor [28]. Ogata et al. report that EGFR-TKI induced severe neutropenia in a case of EGFR-mutated lung adenocarcinoma with chronic myelomonocytic leukemia, suggesting that EGFR-TKI may cause severe hematological side effects in patients with hematological malignancy [29]. In the current case, the combination of gefitinib and nilotinib was safely administered for seven months.

CYP3A4 is the most abundant CYP enzyme in the liver and intestine, and approximately 50% of the currently available drugs are metabolized by CYP3A4 [6]. The drug-drug interaction studies are usually conducted in the course of drug development. However, *in vivo* quantitative data are often lacking for most drug combinations.

We have previously established a quantitative prediction framework of CYP3A4-mediated oral drug interactions. The estimated AUC increases were within a range of 0.5- to 2.0-fold of the observed AUC increases in 57 out of 60 drug combinations [5, 6]. By using this framework, the contribution of CYP3A4 to clearance of EGFR-TKIs after oral absorption can be quantitatively compared between different drugs. For example, osimertinib has lower value of CR_CYP3A4_ than the first generation EGFR-TKIs (gefitinib and erlotinib) (Table 1), and its plasma concentration is predicted to be less influenced by a CYP3A4 inhibitor.

Dasatinib is an inhibitor of CYP3A4 and its IR_CYP3A4_ is calculated to be 0.16 based on the 1.2-fold AUC increase of CYP3A4 substrate simvastatin [30]. Using the equation 1/(1 − CR_CYP3A4_ × IR_CYP3A4_), the AUC of erlotinib is predicted to increase only by 1.07. In agreement with the prediction, the AUC of erlotinib was not affected by dasatinib in the phase I/II clinical trial of dasatinib and erlotinib for advanced non-small-cell lung cancer [26].

In summary, we have reported for the first time the safety and efficacy of dual EGFR and ABL tyrosine kinase inhibitor treatment in a patient with concomitant EGFR-mutated lung adenocarcinoma and BCR-ABL1-positive CML. The combination of gefitinib and nilotinib was safely administered for seven months. The limitation of the present report is that we did not measure the actual plasma concentrations of gefitinib with or without nilotinib. Further reports are needed to establish the safety and efficacy of TKI combination therapy.

## Figures and Tables

**Table 1:** Contribution ratio of CYP3A4 to clearance of EGFR-TKIs after oral absorption (CR_CYP3A4_).

	Calculated CR_CYP3A4_	Standard inhibitor^∗^	AUC fold change by standard inhibitor	Reference
Gefitinib	0.46	Itraconazole	1.78	[13]
Erlotinib	0.40	Ketoconazole	1.68	[14]
Afatinib	0^∗∗^			
Osimertinib	0.20	Itraconazole	1.24	[15]

^∗^IR_CYP3A4_ values for itraconazole and ketoconazole are 0.95 and 1.00, respectively (Ref. [5]). ^∗∗^Substrate for the P-glycoprotein (P-gp). Metabolism by cytochrome P-450 is of negligible (Ref. [16]).
